# Architecture Insight of Bifidobacterial α-L-Fucosidases

**DOI:** 10.3390/ijms22168462

**Published:** 2021-08-06

**Authors:** José Antonio Curiel, Ángela Peirotén, José María Landete, Ana Ruiz de la Bastida, Susana Langa, Juan Luis Arqués

**Affiliations:** Departamento de Tecnología de Alimentos, Instituto Nacional de Investigación y Tecnología Agraria y Alimentaria (INIA-CSIC), Carretera de La Coruña Km 7.5, 28040 Madrid, Spain; angela.peiroten@inia.es (Á.P.); landete.josem@inia.es (J.M.L.); ana.ruiz@inia.es (A.R.d.l.B.); langa.susana@inia.es (S.L.); arques@inia.es (J.L.A.)

**Keywords:** bifidobacteria, fucosidases, glycosyl hydrolases, conserved domains, human milk

## Abstract

Fucosylated carbohydrates and glycoproteins from human breast milk are essential for the development of the gut microbiota in early life because they are selectively metabolized by bifidobacteria. In this regard, α-L-fucosidases play a key role in this successful bifidobacterial colonization allowing the utilization of these substrates. Although a considerable number of α-L-fucosidases from bifidobacteria have been identified by computational analysis, only a few of them have been characterized. Hitherto, α-L-fucosidases are classified into three families: GH29, GH95, and GH151, based on their catalytic structure. However, bifidobacterial α-L-fucosidases belonging to a particular family show significant differences in their sequence. Because this fact could underlie distinct phylogenetic evolution, here extensive similarity searches and comparative analyses of the bifidobacterial α-L-fucosidases identified were carried out with the assistance of previous physicochemical studies available. This work reveals four and two paralogue bifidobacterial fucosidase groups within GH29 and GH95 families, respectively. Moreover, *Bifidobacterium longum* subsp. *infantis* species exhibited the greatest number of phylogenetic lineages in their fucosidases clustered in every family: GH29, GH95, and GH151. Since α-L-fucosidases phylogenetically descended from other glycosyl hydrolase families, we hypothesized that they could exhibit additional glycosidase activities other than fucosidase, raising the possibility of their application to transfucosylate substrates other than lactose in order to synthesis novel prebiotics.

## 1. Introduction

**Figure 1:**
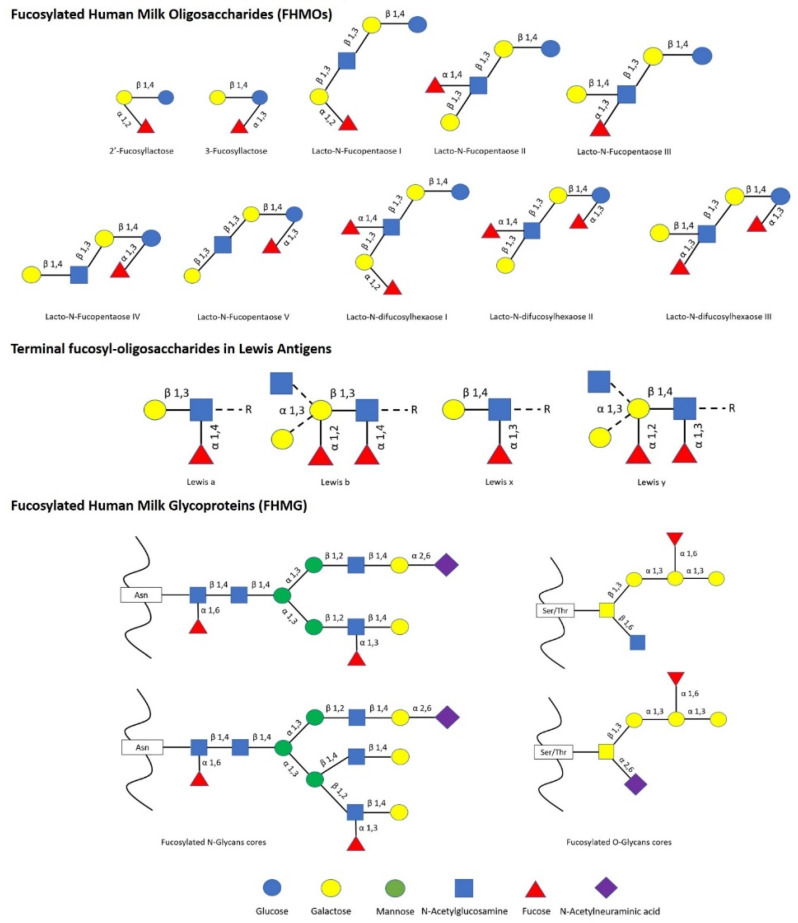
List of main fucosylated human milk oligosaccharides (FHMOs) and fucosylated human milk glycoproteins (FHMG) reported [1,6,7].

The impact of human milk glycobiome on the gut microbiota of infants is well established [1]. While a great part of the components of breast milk provide nutrients to the infant, human milk oligosaccharides (HMOs) and human milk glycoproteins (HMGs) selectively favor the colonization and growth of bifidobacteria in the infant intestine, contributing to the development of the gut microbiota [1,2]. In this regard, *Bifidobacterium* species are considered key actors in the multifaceted process of gut development and maturation of the immune system [3]. In fact, during the first months of birth, the loss of bifidobacteria or the gain of other bacteria can significantly alter the progression of the healthy microbial community with negative consequences for the infant, including a predisposition to autoimmune and/or metabolic diseases such as allergies and childhood obesity [4,5]. Concerning to that, fucosylated HMOs (FHMOs) and fucosylated HMGs (FHMGs) constitute a great part of the glycobiome of the breast milk [6] (Figure 1) and have been proposed to be essential in the development of the microbiota [7].

FHMOs constitute the largest fraction of human milk oligosaccharides, and although they show a small number of different conformations, they can make up to 70% of the total in an individual mother’s milk [6]. The fucosylated trisaccharide 2′-fucosyllactose is the most abundant FHMO, representing from 12 to 45% of the total HMO content in breastmilk, while 3-fucosyllactose is less abundant, from 0.5% to 3% [8]. On the other hand, there are several FMHGs investigated, and contrary to FHMOs, they appear at lower concentration but show a higher number of different forms, including lactoferrin (17%), immunoglobulins IgG (<1%), IgM (<1%), and secretory IgA (11%) [9,10,11]. Both FHMOs and FHMGs stand out for their ability to stimulate the growth of bifidobacteria [7,12], whose metabolism transforms fucosylated oligosaccharides into short-chain fatty acids (SCFAs) such as acetate, formate, lactate, and pyruvate [13], which in turn stimulate the immune system by inducing the differentiation of T-regulatory cells via inhibition of histone deacetylase [14].

The great influence of fucosylated compounds present in breast milk on bifidobacteria is due to their ability to metabolize them, being α-L-fucosidases (henceforth, fucosidases) indispensable tools that allow shaping the gut microbiome in the first months of life.

According to CAZy database, hitherto, more than 10,000 sequences have been identified in silico as α-L-fucosidases, belonging to a wide variety of organisms from archaea to fungi and plants. However, the vast majority of fucosidase sequences have been described in bacteria and belong to more than 2000 bacteria species (www.cazy.org). This database classifies fucosidases into three families (GH29, GH95, and GH151) according to their catalytic structures. GH29 fucosidases act through a retaining mechanism and have a broader substrate specificity, including hydrolysis of Fuc-α1,3/4/6 linkages [15]. Moreover, family GH29 fucosidases have been subclassified into two subfamilies. The subfamily A contains α-fucosidases with relatively relaxed substrate specificities, able to hydrolyze *p*-nitrophenyl-α-L-fucopyranoside (pNP-fucose), while the members of subfamily B are specific to α1,3/4-glycosidic linkages and are practically unable to hydrolyze pNP-fucose [16]. Although GH29 fucosidases also could exhibit hydrolysis of Fuc-α1,2 linkages, that activity is mainly attributed to GH95 family, which catalyzes the hydrolysis of fucose linkages through an inverting mechanism, resulting in the inversion of the anomeric configuration [17,18]. Finally, GH151 family has poor activity on fucosylated substrates; this is the reason why it is currently questioned as to whether they are genuine fucosidases [19,20,21].

Even though species of the *Bifidobacterium* genus dominate the infant gut microbiota in early life, and given the importance of their metabolism of fucosylated conjugates, there are only a few bifidobacterial species studied extensively at both cellular and genomics level for their ability to utilize fucosylated carbohydrates, including *B. bifidum* and *Bifidobacterium longum* subsp. *infantis* [22,23]. However, different strain-dependent metabolic abilities have been unraveled for the use of fucosylated conjugates and are likely determined by their fucosidases’ diversity [24]. Indeed, agreeing with the evolution and phylogenetics of fucosidases previously studied in metazoan fucosidases [25], bifidobacterial fucosidase sequences listed in CAZy reveal substantial in silico differences regarding to their conserved domains, even those ones clustered in the same GH, revealing different adaptation/specialization ranges as well as their origin. Therefore, this work addresses the diverse conserved architectures of bifidobacterial fucosidases and cluster them by activity and phylogenetic evolution in order to propose a novel classification within the GH groups already listed in CAZy.

## 2. Results

### 2.1. Bifidobacterial GH29 Fucosidases

**Figure 2:**
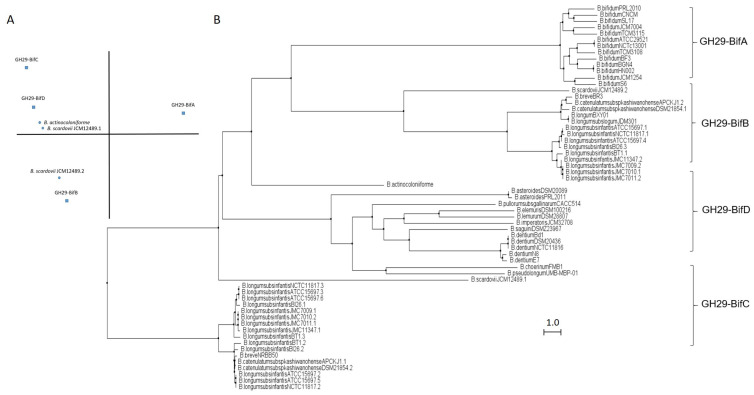
Phylogenetic analysis of bifidobacterial GH29 fucosidases. PCA (**A**) and cladogram tree (**B**) distributions of bifidobacterial GH29 fucosidase sequences listed in CAZy, released from Jalview 2.11.1.4 software using the neighbor-joining method.

GH29 fucosidases from bifidobacteria listed in CAZy are shown in Table S1. Based on in silico studies concerning conserved domains released by NCBI Conserved Domains Database (CDD), bifidobacterial GH29 fucosidases could be classified into four different phylogenetic groups (Table S1). That differentiation was also confirmed through sequence homology PCA and cluster analyses (Figure 2).

The enzymes included in the proposed GH29-BifA, only found in *B. bifidum* strains, are characterized as large membrane-bound fucosidases (AfuC super family domain; NCBI CDD accession number cl34656) and exhibit an accessory F5/F8 type C domain family (NCBI CDD accession number cl23730), probably involved in recognizing galactose or N-acetyllactosamine [26]. Interestingly, while InterPro database (EMBL-EBI) recognized the F5/F8 type C domain (IPR000421), it interpreted the AfuC domain as Glyco_Hydro_29 domain (IPR000933), probably due to the degree of updating of both databases (Table S1). In addition, Ashida et al., 2009 identified a second putative sugar-binding domain in GH29 fucosidase AfcB from *B. bifidum* JCM1254, domain that is frequently found in membrane-bound or cell-wall-associated proteins and denominated FIVAR [27]. Those results were here confirmed by SOSUI and HMMTOP databases, which allowed the identification of two putative transmembrane helices in GH29-BifA fucosidases (Table S1). Therefore, it has been suggested that both accessory F5/F8 type C and FIVAR domains allow the extracellular character of GH29 fucosidases in *B. bifidum* and could enhance affinity toward fucosyl conjugates [27]. Moreover, in all the N-terminal regions of GH29-BifA fucosidases, hydrophobic sequences predicted by SignalP-5.0 to be putative signal peptide with potential cleavage sites were observed (Table S1).

Concerning the AfuC/Glyco_hydro_29 domain, the only representative GH29 fucosidase of GH29-BifA purified and characterized, which is AfcB from *B. bifidum* ATCC 1254, is able to hydrolyze 3-fucosyllactose, Lewis blood group substances (a, b, x, and y types), and lacto-N-fucopentaose II and III. However, the enzyme did not act on glycoconjugates containing α1,2-fucosyl residue or on synthetic pNP-fucose [27].

Supporting the in silico characterization of GH29-BifA fucosidases, several studies confirm the ability of *B. bifidum* to extracellularly hydrolyze FHMOs [28]. However, *B. bifidum* appears to prefer the utilization of lactose when growing on FHMO, probably releasing fucose to the environment [28]. This incapacity to consume fucose may be due to the lack of specific transporters. Nevertheless, the extracellular fucosidase activity of *B. bifidum* could facilitate the establishment of the bifidobacteria community, allowing them to consume the released fucose residues [29].

In contrast to GH29-BifA, the rest of the GH29 fucosidases from bifidobacteria do not have either putative signal peptides or transmembrane helices and consequently their mode of action can be considered intracellular. Indeed, GH29-BifB fucosidases are characterized by exhibiting an AfuC super family/Glyco_Hydro_29 domain (NCBI CDD accession number cl34656/IPR000933) such as GH29-BifA fucosidases but lacking F5/8 type C and FIVAR domains. Due to the presence of the same fucosidase domain in both groups of fucosidases (GH29-BifA and GH29-BifB), similar metabolic capacities could be affirmed. In fact, the only characterized bifidobacterial GH29-BifB fucosidase (Blon_2336 from *Bifidobacterium longum* subsp. *infantis* ATCC 15697) revealed similar activity to AfcB from *B. bifidum* ATCC 1254 (GH29-BifA) against Fuc-α1,3 glucosidic, Fuc-α1,3GlcNAc, and Fuc-α1,4GlcNAc linkages [21]. These GH29-BifB fucosidases appear to be distributed along strains of different species, contrary to GH29-BifA fucosidases, and frequently, strains that exhibit GH29-BifB fucosidases also show GH29-BifC fucosidases, which are duplicated in some of the sequenced strains (Table S1). Actually, the duplication of GH29 fucosidases has been reported previously and plays an important role in fucosidases evolution [30].

GH29-BifC fucosidases are characterized by showing conserved α-Amylase catalytic domain family (NCBI CDD accession number cl38930). It must be taken into account that this superfamily is present in a large number of GHs able to hydrolyze α1,4/6 glycosidic bonds, although in turn they have specific domains unlike the GH29-BifC fucosidases of bifidobacteria [31]. However, since GH29-BifC fucosidases can catalyze the transformation of fucosidic α1-2Gal/3GlcNAc linkages in LNFP I and III, respectively, and mainly Fuc-α1,6 GlcNAc linkages [32], activity non described in the above fucosidase groups, it is difficult to ensure that its catalytic family proposed is α-Amylase catalytic (NCBI CDD) or Glyco_Hydro_29 (InterPro) (Table S1). In this sense, InterPro database (EMBL-EBI) indicated the presence in GH29-BifC fucosidases of a second catalytic family denominated FUC_metazoa_typ (IPR016286) that is close to eukaryotic fucosidases (Table S1). Probably the presence of this domain is key for these fucosidases to be considered as the most unspecific and versatile fucosidases of bifidobacteria since a wide range of substrates has been reported for two different GH29-BifC fucosidases from *B. longum* subsp. *infantis* ATCC 15697 [21,27].

Both GH29-BifB/C fucosidases described in *B. longum* subsp. *infantis* strains are likely found in the cytosol. Therefore, efficient transport of oligosaccharides is needed, unlike *B. bifidum* [13,21]. In this context, genomic studies carried out on *B. longum* subsp. *infantis* ATCC 15697 have unraveled several putative fucose permeases that may facilitate environmental scavenging when soluble fucose is encountered.

In order to elucidate the roles and fitness of the bifidobacterial community to shape the gut microbiome and taking into account the relevance of fucosidases in this regard, their features mentioned above should be updated and expanded to avoid ambiguities in the catalytic domains and relate them to their metabolic properties. Certainly, the rest of the enzymes from different bifidobacterial species need to be characterized in order to reliably distinguish the properties of each group of fucosidases for determining the interaction and mode of actions of bifidobacteria during gut colonization. In this sense, the role of GH29-BifD of fucosidases remains unknown despite having been sequenced and identified in certain *Bifidobacterium* species (Table S1). Unlike to GH29-BifC, GH29-BifD fucosidases exhibit specific α-L-fucosidase main domain (NCBI CDD accession number cl38930). Surprisingly, their accession number is matching with superfamily AmyAc family of group II, suggesting a better accurate and updated in silico annotation. However, InterPro database (EMBL_EBI) indicates both catalytic domain Glyco_Hydro_29 and FUC_metazoa_typ (InterPro IPR000933 and IPR016286, respectively). Nevertheless, physicochemical properties, substrate specificity confirmation, and their correlation with catalytic domains are still pending to be characterized.

### 2.2. Bifidobacterial GH95 Fucosidases

**Figure 3:**
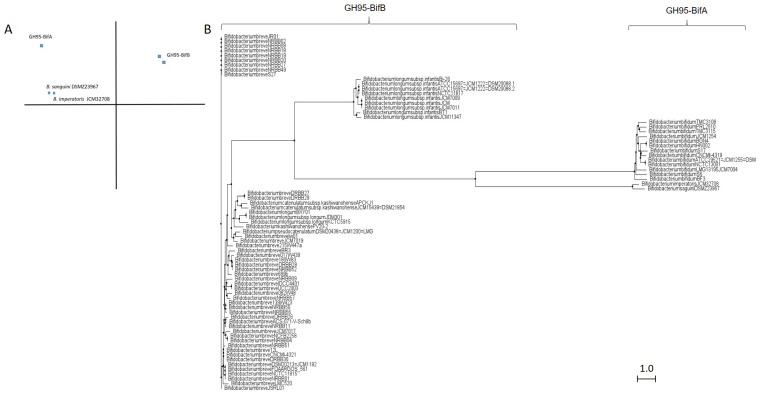
Phylogenetic analysis of bifidobacterial GH95 fucosidases. PCA (**A**) and cladogram tree (**B**) distributions of bifidobacterial GH95 fucosidase sequences listed in CAZy, released from Jalview 2.11.1.4 software using the neighbor-joining method.

Similar to GH29 bifidobacterial fucosidases and according to architecture domains, bifidobacterial GH95 fucosidases collected on CAZy could also be subclassified into two main groups (Table S2; Figure 3).

The extracellular character observed in GH29-BifA fucosidases from *B. bifidum* strains is also reflected in their GH95 fucosidases, which are characterized by a putative signal peptide and two predicted transmembrane helices. Among GH95 fucosidases, those features are only found in the proposed GH95-BifA fucosidases from *B. bifidum* with the exception of *Bifidobacterium saguini* DSMZ 23967 fucosidase (Genbank QTB91571.1), which exhibited two putative transmembrane helices (Table S2).

The proposed GH95-BifA was characterized according to the NCBI CDD database by exhibiting Glycosyl hydrolase 65 N-terminal (accession number cl22392) as main catalytic domain, while InterPro database analysis (EMBL-EBI) revealed a Glycosyl hydrolase 95 N-terminal (IPR027414) (Table S2). The observed ambiguous prediction on the catalytic architecture could be due to the lack of updating and mismatch annotations. Nevertheless, a common evolutionary origin for GH65 and GH95 families, among others, with conservation of their putative catalytic amino acid residues, was noticed and likely influenced the in silico results [18]. Nevertheless, and contrary to GH65 family, the only GH95-BifA representative fucosidase recombinantly produced and characterized (AfcA from *B. bifidum* JCM1254) showed great activity against Fuc-α1,2 Gal linkages, mainly hydrolyzing 2′-Fucosyllactose and lacto-N-fucopentaose I [17,33].

On the other hand, while NCBI CDD database detected two YjdB overlapping domains (accession number cl35007), whose functions are still uncharacterized but in turn contain Ig-like domain, InterPro database noticed Ig-like_Bact and Bacterial Ig-like group 2 (BIG2) domains instead (accession number IPR022038 and IPR003343, respectively) (Table S2). Despite this coincidence, only the position of one domain practically matches in both databases (YjdB and BIG2) (Table S2). In addition, InterPro identifies Ig-like_Bact near to N-terminal unlike NCBI CDD, and probably GH95-BifA sequences could exhibit up to three accessory domains.

It should be noted that, although the function of BIG2 domain has not been unraveled, it has been hypothesized to participate in facilitating the protrusion of the AfcA catalytic GH95 domain from the cell surface to allow its extracellular activity and degrade the fucosyl residues present on glycoconjugates of enterocytes [17]. This fact could lead one to define AfcA as a bifidobacterial tool for protecting the host’s health through modifying α1,2 fucosylated Lewis antigen receptors b and y, recognized by gut pathogens such as *Helicobacter pylori* [34], and norovirus [35]. Taking into account the conserved domains, GH95 fucosidases from *B. imperatoris* and *B. saguini* could be close to being clustered within the GH95-BifA (Table S2). The extracellular character of *B. imperatoris* and *B. saguini* fucosidases could even be affirmed since signal peptides and transmembrane helices are found, although they have not yet been characterized. Indeed, cladogram phylogenetic analysis revealed that both fucosidases actually exhibit more similarities with GH95-BifA (Figure 3).

Beyond GH95-BifA, there are a large number of intracellular GH95 fucosidases from *Bifidobacterium breve* and *B. longum* subsp. *infantis* strains in silico categorized by showing a glycosyl hydrolase 65 N-terminal domain (cl22392; NCBI CDD). They share the catalytic domain with GH95-BifA without exhibiting accessory BIG2 (Table S2). Nevertheless, InterPro database managed to identify a catalytic domain of greater length than in the GH95-BifA sequences, denominated Alpha_L_Fuco family (IPR016518). The presence of this domain could be the key for *B. breve* and *B. longum* subsp. *infantis* GH95 fucosidases to show phylogenetic differences with GH95-BifA as shown by the PCA and cladogram analyses (Figure 3), and therefore are clustered in GH95-BifB.

Unfortunately, no *B. breve* GH95-BifB fucosidases have yet been characterized, although the described hydrolytic activity of *B. breve* on Fuc-α1,2 Gal linkages supports the presence of a functional GH95 fucosidase [36]. Blon_2335 from *B. longum* subsp. *infantis* is the only representative of GH95-BifB that has been characterized [21]. In that study, Blon_2335 showed a strong preference for Fuc-α1,2 linkages (2′-FL, LNFP-I), although it partially cleaved Fuc-α1,3 linkages (3-FL), unlike AfcA from *B. bifidum* [21]. Because AfcA structural exploration revealed its catalytic reaction as a α1,2 fucosidase [18], and since both AfcA and Blon_2335 fucosidases show catalytic architecture differences, further studies concerning crystallization of Blon_2335 are needed in order to elucidate its ability for hydrolyzing both Fuc-α1,2 and Fuc-α1,3 linkages. Structure elucidation could also explain the substantial differences between the GH95-BifB fucosidases from *B. breve* and *B. longum* subsp. *infantis*, also observed in PCA and cladogram (Figure 3), despite presenting the same conserved architecture (Table S2).

### 2.3. Bifidobacterial GH151 Fucosidases

**Figure 4:**
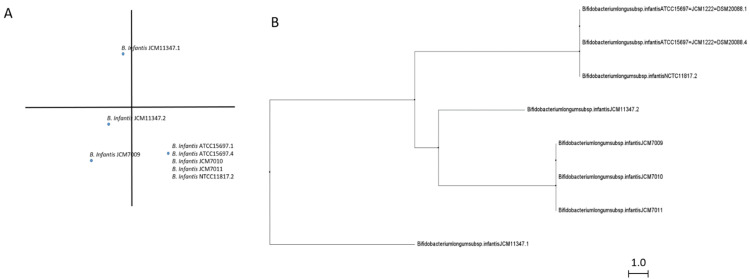
Phylogenetic analysis of bifidobacterial GH151 fucosidases. PCA (**A**) and cladogram tree (**B**) distributions of bifidobacterial GH151 complete fucosidase sequences listed in CAZy, released from Jalview 2.11.1.4 software using the neighbor-joining method.

GH151 enzymes form the smallest group of fucosidases (Table S3) and although there are still doubts about their fucosidase activity, *B. longum* subsp. *infantis* ATCC 15697 counts, with a GH151 enzyme (Blon_0346) that exhibits probed Fuc-α1,2 Gal activity [21]. Interestingly, bifidobacterial GH151 fucosidases are quite divergent from the fucosidases classified in other GH families [21] and all of them belong to *B. longum* subsp. *infantis* species although they show little differences in their sequences (Figure 4). While no signal peptide or transmembrane helices were observed, CDD architecture analyses revealed AmyAc_family superfamily and A4_beta-galactosidase_middle_domain, although some sequences are also identified as containing GanA superfamily domain as well (Table S3).

GH151 enzymes probably have domains closest to GH29-BifC fucosidases, identified by containing conserved AmyAc superfamily domain and likely the ability to hydrolyze α glycosidic linkages [31]. However, because GH151 accessory domains shown (Table S3), they could be considered as potential non-specific beta galactosidase enzymes with the capacity to hydrolyze Fuc-α1,2 Gal linkages as occurs with Blon_0346. Nevertheless, further studies in order to elucidate their subjacent activity, substrate specificity, and conformational structure are needed to understand their role in the hydrolysis of fucosylated carbohydrates.

## 3. Discussion

Breast milk, beyond its nutritional function, provides the necessary pillars for the initial establishment of the gut microbiota in newborns. In this regard, FHMOs and FHMGs stand out for their ability to stimulate the growth of bifidobacteria [8,12], which in turn produce SCFAs such as acetate, formate, lactate, and pyruvate [13], stimulating the immune system [14], and serving as an energy source for colonocytes [37].

Although only a few bifidobacterial species have been studied extensively at both cellular and genomics level for their ability to utilize fucosylated carbohydrates such as *B. bifidum* and *B. longum* subsp. *infantis* [22,23], their success in colonizing the gut is due to the different strain-dependent metabolic abilities developed for the use of both FHMOs and FHMGs [24]. Therefore, fucosidases play a key role in the bifidobacterial gut establishment. Concerning to that, *B. bifidum* strains show two extracellular fucosidases belonging to GH29 and GH95 families. Both fucosidases cover the hydrolysis of Fuc-α1,3Glu; Fuc-α1,3/4GlcNAc; and Fuc-α1,2 Gal linkages [17,18,27,33]. Since *B. bifidum* prefers the utilization of lactose [28], 2′-fucosyllactose could be its target substrate for its extracellular fucosidases, releasing to the environment lactose and fucose, the last could be also liberated from blood Lewis a, b, x, and y antigens [27]. For all the above, *B. bifidum* fucosidases could be considered altruistic and essential for microbial gut establishment through promoting bifidobacterial mutualism and carbohydrate syntrophy in the infant gut [38]. Given that bifidobacteria are able to metabolize lactose, and species such as *B. longum* subsp. *infantis* or *B. breve* can metabolize fucose, their growth is improved under the presence of fucosidases from *B. bifidum*. Thus, Gotoh et al. (2018) suggested that extracellular fucosidases from *B. bifidum* could be crucial during the development of a bifidobacteria-rich microbiota in the breastfed infant gut, by providing fucosylated conjugate degradants [33]. On the other hand, *B. bifidum* fucosidases contribute to the protection of the host through the modification of Lewis antigens [27].

Regarding the catalytic domains of the *B. bifidum* fucosidases, it should be noted that GH29-BifA present orthologous fucosidases in other bifidobacterial species clustered in GH29-BifB/D, and they probably all have a common phylogenetic lineage (Figure 2). However, this statement has only been functionally corroborated through the characterization of the enzymes AfcB (GH29-BifA) and Blon_2336 (GH29-BifB), due to lack of results of GH29-BifD fucosidases.

Conversely, GH95-BifA fucosidases as well as those grouped in GH95-BifB, and according to CDD database observations (Table S2), could phylogenetically descend from either an evolutionary specialization or non-specification of glycosidases clustered in GH65. Indeed, this in silico observation agrees with the crystallization results obtained for the structure AfcA from *B. bifidum* [18]. According to that, both GH65 and GH95 enzymes share an α/α 6 barrel fold with inverting mechanism and glutamate^566^ as catalytic proton donor. Moreover, Nagae et al. (2007) compared the structures between families GH65 and GH95, revealing conservation of the general acid residues, except for catalytic acid/base aspartate^766^, which is shifted in AfcA [18]. That shifting was also found in the rest of the bifidobacterial GH95 fucosidases (data not shown), and agreeing with the above mentioned authors, the reaction mechanisms of bifidobacterial GH95 fucosidases differ from those of the GH65 family [18].

The other species widely studied for its fucosidase activity is *B. longum* subsp. *infantis*. Actually, it is the only species of bifidobacteria that exhibits GH29, GH95, and GH151 fucosidases that have been recombinantly purified and characterized [21]. Those fucosidases allow *B. longum* subsp. *infantis* to use a wide range of substrates, hydrolyzing Fuc-α1,3Glu; Fu-cα1,2/3Gal; and Fuc-α1,3/4/6GlcNAc linkages [21,32]. As previously commented, *B. longum* subsp. *infantis* GH29-BifB fucosidases are orthologous with those classified in GH29-BifA. However, this species also shows GH29-duplicated fucosidases, clustered in the GH29-BifC, with different architecture and paralogs from those of GH29-BifB (Figure 3). Taking into account the fucosidase duplication and in agreement with You et al. (2019), *B. longum* subsp. *infantis* GH29-BifC fucosidases could have evolved from a different glycosyl hydrolase [30]. According to CDD database observations (Table S1) and because their predicted structure is composed by a β/α 6 barrel fold with retaining mechanism and glutamate as catalytic proton donor, GH29-BifC fucosidases from *B. longum* subsp. *infantis* could descend from GH13 glycosidases (α-amylases).

GH29-BifC fucosidases, similar to GH95-BifB, which is probably phylogenetically originated from GH65 family as described above, need to have their structural crystallization further explored in order to elucidate their origins and evolution pathway. In addition, GH29-BifC fucosidases show similarities with metazoan fucosidases according to the InterPro database (Table S1), including aspartate^224^ and glutamate^270^ residues (data not shown), which play the role of the catalytic nucleophile and catalytic acid/base, respectively, in metazoan fucosidases [25].

Finally, GH151 fucosidases are exclusively present in *B. longum* subsp. *infantis*. This fact could suggest a fourth pathway of fucosidases phylogenetic evolution in that species closely related to GH29-BifC fucosidases, since they present a N-terminal α amylase catalytic domain. In addition, Blon_0346 was originally classified as a member of GH29 family due to their fucosidase activity despite low similarity [21]. However, GH151 enzymes may be the result of a branch in the evolution of GH29-BifC fucosidases, since they show a GH42 beta galactosidase trimerization architecture instead of conserved features of metazoan fucosidases.

## 4. Materials and Methods

### 4.1. Identification and Selection of Fucosidase Sequences

Complete bifidobacterial fucosidase protein sequences belonging to GH29, GH95, and GH151 families were retrieved from CAZy database [19]. Fucosidase sequences were used as probes in PSI-BLAST searches [39] against the NCBI [40], Swiss-Prot [41], and Ensembl [42] protein databases.

### 4.2. Protein Sequence, Alignment, and Phylogenetic Analysis of α-L-Fucosidases

Fucosidase sequences were analyzed using SignalP-5.0 [43], with default options to predict signal peptide sequences: SOSUI [44] and HMMTOP [45] with default parameters for the prediction of transmembrane helices. NCBI Conserved Domains Database (CDD) [46] and InterPro databases (EMBL_EBI) [47] were used to predict the domain architecture. Inferred fucosidase amino acid sequences were aligned using Clustal Omega web version [48]. All sequences belonging to the same GH families were considered in phylogenetic analyses. Neighbor-joining method cladogram and PCA analyses were performed using the program Jalview 2.11.1.4 [49].

## 5. Conclusions

This is the first study that explores phylogenetically the three families of the bifidobacterial fucosidases: GH29, GH95, and GH151, through their conserved architecture, showing that *B. bifidum* and *B. longum* subsp. *infantis* reveal two and four different phylogenetic lineages, respectively, belonging to different fucosidase families. On the other hand, given the differences in the catalytic architecture observed in this work, the bifidobacterial fucosidases belonging to the GH29 and GH95 families could be subclassified into four and two groups, respectively.

Taking into account that the observations described in this work were obtained in silico and supported by current characterization results from some *B. bifidum* and *B. longum* subsp. *infantis* fucosidases, further studies regarding structural characterization and physicochemical properties of more fucosidases identified by computational analysis are needed in order to validate the novel classification of bifidobacterial fucosidases here proposed.

Concerning to *B. longum* subsp. *infantis* fucosidases, which evolved from different GH families such as GH29-BifC, GH95-BifB, and GH151, and given that their conserved architecture presents vestiges of ancestral glycosidases GH13, GH65, and GH42, respectively, as well as *B. Bifidum* GH95-BifA fucosidases phylogenetically descended from GH65, deepening substrate spectrum analyses could determine their underlying roles in those species. In this context, and since some fucosidases have been used to transfucosylate carbohydrates or glycoconjugates, the application of these evolved and hypothetically non-specific *B. longum* subsp. *infantis* fucosidases mentioned above can open a new perspective towards the synthesis of novel fucosylated conjugates by using different substrates beyond lactose for synthetizing 2′-fucosyllactose. This vision is oriented towards the supply those novel fucosylated conjugates to adults in combination with fucosidase producer bifidobacteria in order to maintain a healthy microbiota or to reestablish it from dysbiosis states as described previously [50,51]. In this regard, it would be important to elucidate phylogenetically, as well as structurally and physicochemically, the fucosidases of many other gut microorganism genera, as for instance *Lactobacillus, Bacteroides,* and *Akkermansia*, with the aim to reveal the whole gut fucosidase interaction.

## Data Availability

Not applicable.

## Supplementary Materials

The following are available online at https://www.mdpi.com/article/10.3390/ijms22168462/s1.

## Abbreviations

FHMG	Fucosylated human milk glycoprotein
FHMO	Fucosylated human milk oligosaccharide
Fuc	Fucose
Gal	Galactose
GH	Glycosyl hydrolase
GlcNAc	N-acetylglucosamine
Glu	Glucose
HMG	human milk glycoprotein
HMO	human milk oligosaccharide
LNFP	Lacto-N-Fucopentaose
pNP-fucose	*p*-nitrophenyl-α-L-fucopyranoside
SCFAs	Short-chain fatty acids.
